# The effect of season and post-fire on habitat preferences of the endangered Swayne’s hartebeest (*Alcelaphus buselaphus swaynei*) in Maze National Park, Ethiopia

**DOI:** 10.1186/s12898-020-0275-3

**Published:** 2020-01-28

**Authors:** Misganaw Tamrat, Anagaw Atickem, Diress Tsegaye, Paul Evangelista, Afework Bekele, Nils Chr. Stenseth

**Affiliations:** 1 Centre for Ecological and Evolutionary Synthesis (CEES), Department of Biosciences, University of Oslo, Blindern, PO Box 1066, 0316 Oslo, Norway; 2 Department of Zoological Sciences, Addis Ababa University, PO Box 1176, Addis Ababa, Ethiopia; 3 Department of Biosciences, University of Oslo, Blindern, PO Box 1066, 0316 Oslo, Norway; 4 Faculty of Environmental Sciences and Natural Resource Management, Norwegian University of Life Sciences, PO Box 5003, 1432 Ås, Norway; 5 Natural Resource Ecology Laboratory, B254 NESB, Colorado State University, Fort Collins, CO 80524-1499 USA

**Keywords:** Fire, Grassland habitat, Grass height, Habitat preference, Swayne’s hartebeest

## Abstract

**Background:**

The availability of preferred habitats determines the spatial and temporal distribution of herbivores in savanna ecosystems. Understanding habitat preference of a targeted wildlife species is crucial for developing effective conservation strategies. Habitat preference of large grazers in connection to grass height and post-fire effect has been debated for the last century. Here, we examined the effects of season, grass height and burning on the habitat preference on Swayne’s hartebeest (*Alcelaphus buselaphus swaynei*) in Maze National Park. Data for seasonal habitat selection were collected using both direct observation along established transect lines and pellet counting using permanently established plots. Every month, we measured grass height commonly preferred by Swayne’s hartebeest in grassland habitat. Starting from the first week of burning, we recorded the abundance of Swayne’s hartebeest in both burned and unburned grassland patches.

**Results:**

From detected pellets, 94.3% were recorded in the grassland habitat indicating that other habitat types are less used despite their extensive cover > 50% of the Park. During wet and early dry seasons, Swayne’s hartebeest exclusively preferred grassland habitat. We found that 85.2% (n = 1079) and 85.3% (n = 593) of individuals observed in areas with a grass height below 30 cm during wet and early-dry seasons, respectively; while 70.9% (n = 2288) preferred grass height below 30 cm during the dry season. The density of Swayne’s hartebeest in burned grassland area was higher than unburned grassland areas up to 150 days since burning. However, in unburned grassland areas, the density was initially low but showed increasing trend for consecutive days, reaching similar density with burned areas after 150 days since burning.

**Conclusion:**

Swayne’s hartebeest exclusively preferred grassland habitat, particularly during wet and early-dry seasons, shortest available grass height in all seasons and were attracted to burned grassland areas. Our results suggested that fire played an important role in maintaining habitat quality in grassland, and that management should continue using controlled burning as a tool for the conservation of Swayne’s hartebeest. However, we remain cautious of our findings given the paucity of information regarding other confounding factors and the absence of long-term data on fire disturbance.

## Background

Identifying the quality and preference of different habitat types are crucial for developing conservation strategies of a targeted wildlife species [1–5]. Herbivores are known to select habitats that provide maximum forage intake [6, 7], while reducing predation risk [5, 8]. There are several factors that can determine the spatial and temporal distribution of herbivores in savannas ecosystems. These include the availability of resources [3, 9–12], predation risk [5, 8], fire [13–15], vegetation height and cover [16–18], human presences and livestock density [19–21]. Since a habitat type may not always have adequate resources, the trade-offs between costs and benefits associated with searching and utilizing forage can limit herbivore selection [22]. Moreover, spatial variation in relative availability of different habitat types may result in dissimilar habitat selection among individuals of the same species [23, 24].

In savanna grassland, where there is a cyclic rainfall, fire is used as one of the most important habitat management tool for herbivores [2, 14, 25, 26]. Understanding how wildlife species respond to fire effects is crucial, particularly for endangered species that have limited range [15, 26]. Fire effects grass height, which in turn effects habitat preference of herbivores [25, 27]. Previous studies [17, 22, 23] have identified the trends of large grazers’ habitat preference in response to grass height and post-fire effect on vegetation. Herbivores could optimize their daily forage need where they are able to access the preferred grass heights [28, 29]. There is a general consensus that grass height has a major influence on the spatial and temporal distribution of herbivores, and resource partitioning among herbivores could also occur through differential selection of grass height [30, 31]. The grass height has been demonstrated to exert a major influence on bite size that in turn impacts on food intake rate achieved by grazing herbivores [24, 28, 32].

Larger body sized herbivores (> 100 kg body weight) [22, 33] are expected to graze taller grasses to meet their quantitative food requirements [18, 34], while smaller body-sized herbivores can achieve an adequate amount of food intake from short grass swards [35, 36]. In theory, shorter grasses are generally leafy with higher proportion of nutrients and preferred by many small body-sized herbivores [22], while larger body-sized herbivores can tolerate poorer quality food provided by the taller grasses [18, 33]. When grass grows and matures, its nutritional quality decreases [9, 18, 32, 37]. This can be demonstrated by the decrease proportion of leaves and the nitrogen content (both indicating high grass quality) in the grass with increasing grass mass in savanna ecosystem [38].

In African savannas, frequent burning of grass influences the habitat selection of herbivores due to impacting forage quality and reducing predation risk [15, 39], and it is a key element in predicting habitat selection by specific species [24]. Fire plays a determinant role in the ecology and evolution of grassland ecosystems [13, 40, 41], and has historically, and still today, been used as a tool for managing grassland vegetation [27, 42]. Post-fire regrowth of grass influences the dry season habitat use of many herbivore species [27, 43]. However, there have been arguments among ecologists how burning affects habitat selection of large body-sized herbivores.

Small body-sized herbivores might prefer burned areas more than large body-sized herbivores due to differential preferences in relation to forage quality [44]. However, another study revealed that fire does not have relationship between body size and use of burned areas [23]. Several studies [14, 23, 45] found that decreasing fire frequency increases vegetation cover and tree densities, which in turn decreases visibility and the subsequent ability of herbivores to detect and escape from predators. As a result, herbivores may avoid areas with relatively denser vegetation cover or spend more time in those areas for vigilance rather than foraging [14]. Hence, herbivores foraging in burned areas may represent either acquiring quality forage or avoiding predators.

Swayne’s hartebeest (*Alcelaphus buselaphus swaynei*) is a large body-sized herbivore weighing between 100 and 200 kg [46]. It was once widely distributed in Ethiopia, Somalia and Djibouti [47], but currently its range is confined in two protected areas: Senkele Swayne’s Hartebeest Sanctuary and Maze National Park in Ethiopia [46, 48, 49] and listed as endangered sub-species by IUCN Red list [50]. In our recent study, we documented the largest population of Swayne’s hartebeests in Maze National Park (Misganaw et al. unpublished), which remains unstudied and receives little attention from the scientific community. Seasonal burning is used as a habitat management tool in the Park, but how the Swayne’s hartebeests respond to post-fire effect and grass height preferences in different seasons remain untouched. Despite its small area, the Park has different habitat types [46]. While hartebeest are known to be grazers [51, 52], there may be conditions that enforce Swayne’s hartebeests to utilize bushland and forest habitats in different seasons. Therefore, the aim of this study was to examine: (1) the extent of different habitats used by Swayne’s hartebeest, (2) the grass height preference of Swayne’s hartebeest, (3) the density of Swayne’s hartebeest in different seasons, and (4) how Swayne’s hartebeest respond to post-fire effect in consecutive days since burning in grassland areas.

## Methods

### Study area

**Fig. 1:**
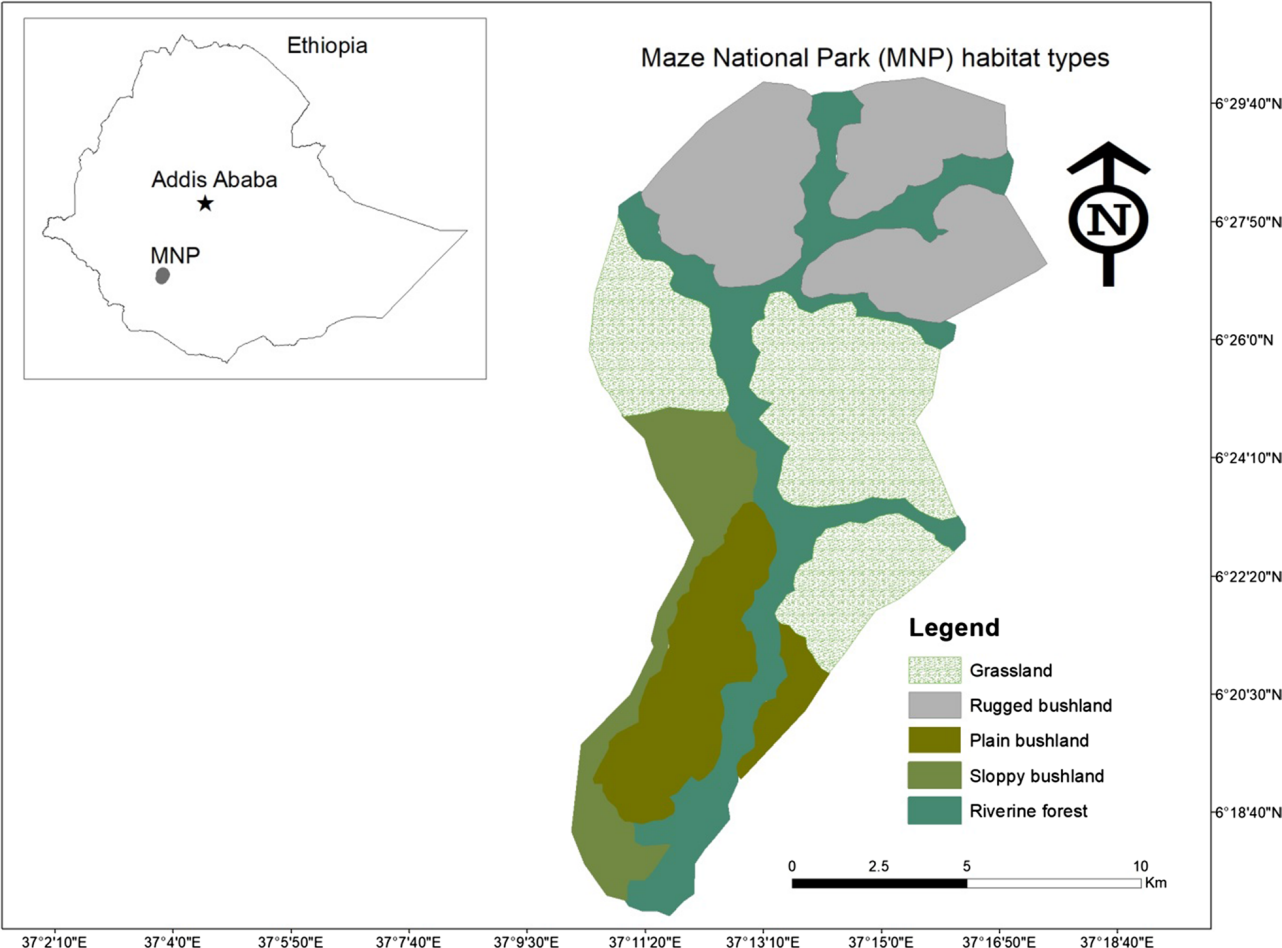
Map showing the study area and habitat types in Maze National Park, Ethiopia (this map was developed by Misganaw Tamrat using ArcMap 10.7.1)

Maze National Park is located at 6°25′N, 37°14′E in southern Ethiopia (see Fig. 1). The Park covers an area of 175 km^2^ and was established in 2005 to conserve the rare and endangered Swayne’s hartebeest, which is considered a flagship species for the Park. The elevation of the study area ranges between 900 and 1300 m asl. It is semi-arid and drought prone area with low and erratic rainfall (mean annual rainfall is below 800 mm) with high mean monthly temperature not less than 30 °C. The Park has sufficient water sources for wildlife. The Maze River and several small tributaries, such as Daho, Lemasea and Domba flow throughout the year in the Park.

Maze National Park has a variety of habitat types, including riverine forests, plain grassland habitats with scattered trees (hereafter called grassland), steep bushland habitat above 15° slope (hereafter sloppy bushland habitat, see Additional file 1), plain bushland habitat, riverine forest, rugged bushland habitat with small valleys and neighborhood agricultural land (Fig. 1). Mountains, agricultural land and communal grazing lands surround the Park. The grasslands are primarily dominated by annual grass species, such as *Exotheca abyssinca*, *Heteropogon contortus*, *Loudentia* spp., *Setaria incrassate*, and *Hyparrhenia filipendula* with scattered woody plants such as *Combretum terminalia*. Burning of the grassland patches have been controlled by the wildlife managers since the Park was established.

### Swayne’s hartebeest sampling design

We initially divided Maze National Park into 10 blocks using features such as roads, rivers, vegetation cover and valleys for a total count of Swayne’s hartebeest in each of habitat types and burned/unburned grassland patches. In each block, the habitat types and burned/unburned grassland patches were demarcated by using GPS within approximately 30 m accuracy and the extent was estimated using ArcGIS 10.3. In each block, we established permanent parallel transect lines spaced approximately 150–200 m apart. In the plain areas (i.e. open grassland and plain bushland areas), 37 transect lines were spaced at 200 m gap, whereas in the forest and rugged bushland areas where observation from distance was impossible, 15 transect lines were spaced 150 m apart. The length of transects varied according to the size of each habitat types with average length of 5.9 km (± 1.5 SD). We established plots (4 m × 5 m) systematically along each transect lines at every 100 m regular intervals (thus, the total is 10 plots per 1 km) for Swayne’s hartebeest pellet presence/absence detection. A total of 1002 plots (i.e., 400 in the grassland, 100 in the plain bushland, 119 in the sloppy bushland, 191 in the rugged bushland, 148 in the riverine forest habitat and 44 in the agricultural land adjacent to the Park boundary) were permanently established. The GPS coordinates and habitat types were recorded at each plot.

### Swayne’s hartebeest habitat selection

The general habitat use of Swayne’s hartebeest from the available six habitat types were conducted for one year (i.e. from December 2016 to November 2017). Since the grass height varied before and after burning the grassland habitat, we divided the dry season into early-dry season (before burning) and dry season (after burning). During the dry season (i.e. from December to May), we counted the pellet samples across the 1002 plots established in the whole Park. Pellet-groups that were more than 50 cm apart in a plot were recorded as pellet from different individuals. We visited each plot for an average of 36 times during the dry season. After a pellet-group was recorded, it was removed from each plot to avoid being recorded during the subsequent surveys.

In the wet (i.e. from June to August) and early-dry seasons (i.e. from September to November), we used direct observation of Swayne’s hartebeest along transect lines as pellet sampling was difficult due to dense habitat cover. During both seasons, habitat use of the Swayne’s hartebeest was estimated through transect counting aided with 10 × 42 binoculars. Whenever the Swayne’s hartebeests were observed, habitat types and abundance of the Swayne’s hartebeests were recorded [39]. We surveyed each transect 12 times during each season, and to avoid double detections of individuals, all transect lines of a block were surveyed at the same time. The surveys were carried out at early morning from 6:00 to 10:00 a.m. and late evening from 3:00 to 6:00 p.m. when Swayne’s hartebeests were active [62].

Because Swayne’s hartebeests were found in three concentrated patches in grassland habitat during the wet season, we delineated the area by using GPS coordinates with 30 m intervals resulting 0.7 km^2^, 2.3 km^2^, and 2.5 km^2^ (see Additional file 2). We also found that the Swayne’s hartebeests shifted in the three peripheral part of the Park during the early-dry season, which covered 3.4 km^2^, 4.7 km^2^ and 5.3 km^2^ areas (see Additional file 2). The density was then derived by dividing the population estimate of the Swayne’s hartebeest during the transect count to the area where they found in the wet and early-dry season.

### Swayne’s hartebeest grass height preferences

To estimate average grass height in the grassland habitat during each season, grass heights were measured for 464, 193 and 133 central points of random plots of one m^2^ area during the dry, wet and early-dry seasons, respectively. The average grass height was varied across seasons in the Park. From the randomly measured grass heights, overall grass height for the survey year was 56.8 ± 60.4 cm (mean ± SD); while for dry, wet and early-dry seasons was 32 ± 39.9 cm, 70.2 ± 51 and 121.7 ± 76 cm, respectively. Based on this estimate, we subjectively categorized the grass heights as below 30 cm, 31–50 cm, 51–100 cm, and above 100 cm.

During the three seasons, the grazing events of Swayne’s hartebeests were recorded to determine the grass height preferred by Swayne’s hartebeest by walking on the transect lines established in the grassland areas. The surveys were carried out for 5–8 days in every month for 1 year (i.e., from December 2016 to November 2017). Whenever an individual or herd of Swayne’s hartebeests was observed on the transect walk within 150 m of either side of a transect line (i.e., 300 m width) for open habitats (i.e., grassland, plain bushland, sloppy bushland and agricultural land), and within 100 m (i.e., 200 m width) for riverine forest and rugged bushland habitats, first their abundance was recorded. Then their feeding location was identified using the nearby landmarks like trees or bushes. The Swayne’s hartebeests were then displaced and fresh bites were identified at the site using the landmarks. Fresh bites were identified by the white coloration at the bite, whereas old bites turn brown [39]. Once the bites were identified, one m^2^ quadrat was placed over the grass patch. Within each quadrat, heights of the preferred grasses by Swayne’s hartebeest were measured, but only those escaped from fresh grazing during the observation time.

### Effect of fire on Swayne’s hartebeest habitat use

The Maze National Park management conducted controlled burning on some parts of the grassland habitat at the end of the wet season every year (mostly from October to November, depending on when the rain ends). Only some portion of the grassland habitat is burned in every year. Burning practice in the Park is mainly maintained by the Park managers with scheduled time in a year for herbivores use. However, in some places mostly at the periphery the local farmers also conduct burning. During this study period, the burning time was end of November, and 21.4 km^2^ of the grassland area was burned while 30.2 km^2^ remained unburned. In both habitat types, we carried out 36 times transect count (a transect might cross both grassland types) from the first date of burning (i.e. from the beginning of December—to mid-May and recorded the abundance of Swayne’s hartebeests in both areas). In both grassland areas, we counted the Swayne’s hartebeests twice (two days) every week to examine how long Swayne’s hartebeests were attracted in those areas. We summed the number of observed individuals for each surveying days in the burned and unburned grassland areas, separately. Counting was conducted in the morning 6:00–10:00 a.m. and late evening from 3:00 to 6:00 p.m. [62].

### Data analysis

#### General habitat use

We used Ivlev’s selectivity calculations as a measure of relative habitat selection of Swayne’s hartebeest among the different habitat types using pellet presence data. Following [39], we used the equation *Ei *=* (ri* − *ni)/(ri *+ *ni)* where *ri* is the proportion of pellet detected in each habitat types within the survey period and *ni* is the proportion of plots in each habitat types during the surveying period available from the total area represented by the survey period.

We used linear mixed effect model from the package lme4 [63] to evaluate the relationship between density of Swayne’s hartebeest pellet (response variable) and habitat types during the dry season. We also used linear mixed effect model to evaluate the relationship between density of Swayne’s hartebeest (response variable) and time (i.e. Julian date as explanatory variable) during early-dry and wet seasons, separately. We used generalized linear model to estimate the relationship between grass height (response variable) and Julian date (explanatory variable) for one year. We also added a squared term for Julian date since it showed a curvilinear trend. We used generalized linear mixed model for Swayne’s hartebeest seasonal grass height preference using density of Swayne’s hartebeest as a response variable with season (at three levels: wet, early-dry and dry) and grass height as predictor variable. Block and transects were used as random factors to account for variations among areas and transects for the above models [64]. We also used generalized linear mixed model to estimate Swayne’s hartebeest abundance (response variable) in relation to burning (categorical variable at two levels: burned and unburned), and days since burning as predictor variable. Block was used as random factor to account for variations among areas [64]. We checked residuals and all the models met the assumption of normality. All analyses were done in R version 3.5.1 [65].

## Results

### Habitat selection

**Fig. 2:**
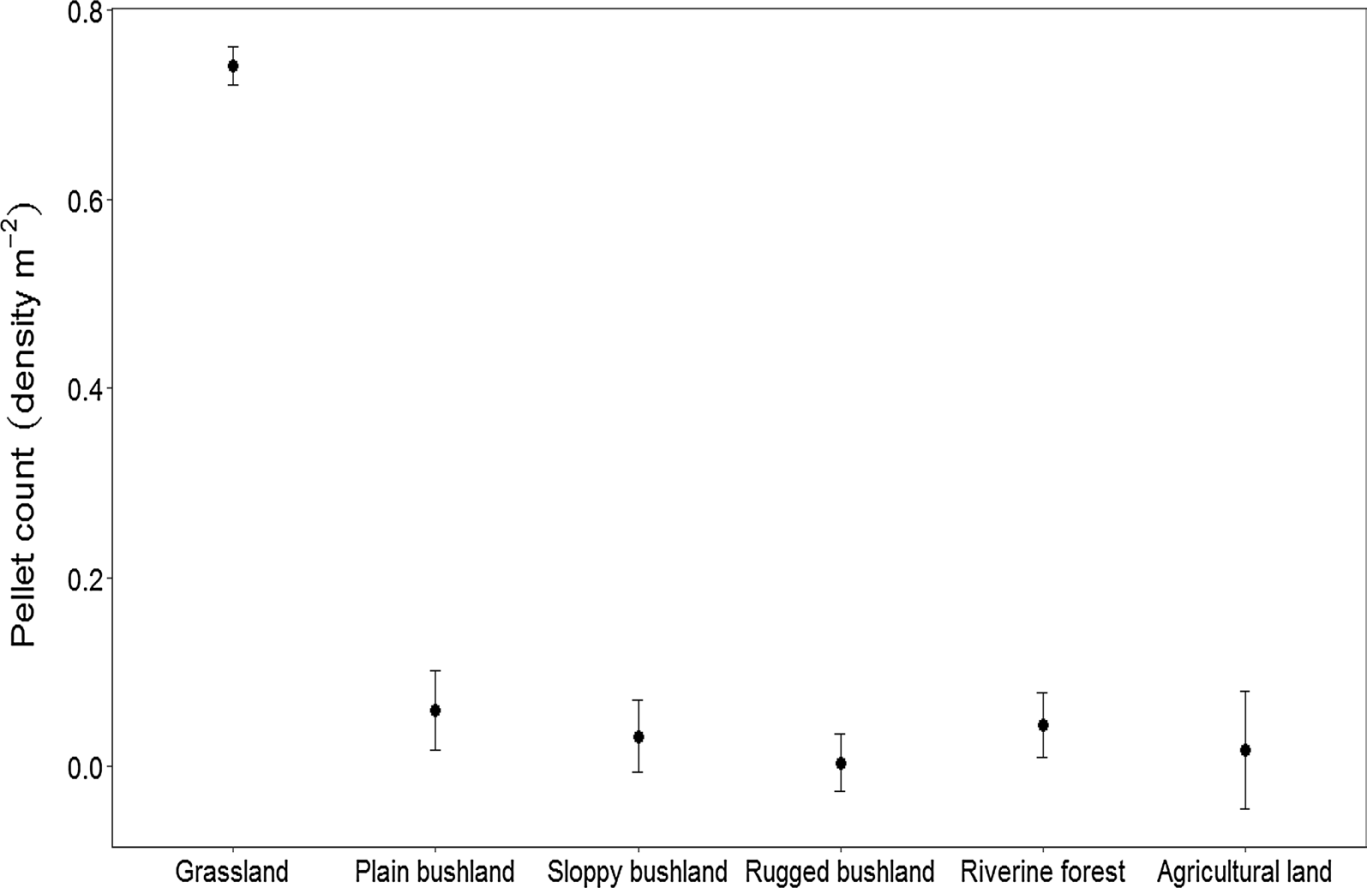
Swayne’s hartebeest pellet density per square meter area in different habitat types during the dry season in Maze National Park

During the dry season, we recorded 6288 Swayne’s hartebeest pellets. Of this, 5931 (94.3%) were in the grassland habitat, 131 (2%) in the riverine forest, 119 (1.9%) in the plain bushland habitat. The rest 107 (1.7%) pellets were found in sloppy bushland, rugged bushland and neighboring agricultural areas. Swayne’s hartebeests selected grassland habitat, while avoiding the remaining five habitats (Table 1). Additionally, the grassland habitat had a significantly higher pellet density than the other habitat types (Fig. 2).

**Table 1:** Number of permanent plots established along the transect routes and the number of pellets detected during the dry season

Habitat type	Number of plots	Number of pellets detected	*r*_*i*_	*n*_*i*_	*E*_*i*_=* (r*_*i*_ − *n*_*i*_*)/(r*_*i*_ +*n*_*i*_*)*
Grassland	400	5931	0.90	0.4	0.40
Plain bushland	100	119	0.02	0.10	− 0.67
Sloppy bushland	119	76	0.01	0.12	− 0.83
Rugged bushland	191	16	0.00	0.19	−1.00
Riverine forest	148	131	0.02	0.15	− 0.76
Agricultural land	44	15	0.00	0.13	− 1.00
Total	1002	6288			

One plot is 4 × 5 m = 20 m^2^; *ri* = is the proportion of all Swayne’s hartebeest pellet detected; *ni* = is the proportion of plots representing a habitat type; *Ei* = Ivlev’s selectivity index

We recorded a total of 154 and 93 of either an individual or herds of Swayne’s hartebeest observation points during wet and early-dry seasons, respectively. All observations were exclusively recorded in grassland habitats. We did not observe Swayne’s hartebeests in other habitat types, because they did not use other habitat types except grassland in both seasons. Of those observation points, we recorded 1269 and 723 Swayne’s hartebeests during wet and early-dry seasons, respectively. During the wet season, no monthly variation on density (individuals/km^2^) of Swayne’s hartebeest was found. However, during the early-dry season the Swayne’s hartebeests were more dispersed to the periphery and the density showed significant decrease with increasing time across months (Table 2).

**Table 2:** Swayne’s hartebeest density (/km^2^) during the wet and early dry seasons in Maze National Park analysed using linear mixed effect model

Season	Effects	Estimate	SE	t-value	p-value
Wet^a^	Intercept	48.277	26.183	1.844	0.070
	Julian date	− 0.169	0.132	− 1.287	0.209
Early dry^b^	Intercept	27.624	8.788	3.143	0.003
	Julian date	− 0.065	0.027	− 2.412	0.020

^a^AIC = 552.015

^b^AIC = 473.120

### Grass height preferences

**Fig. 3:**
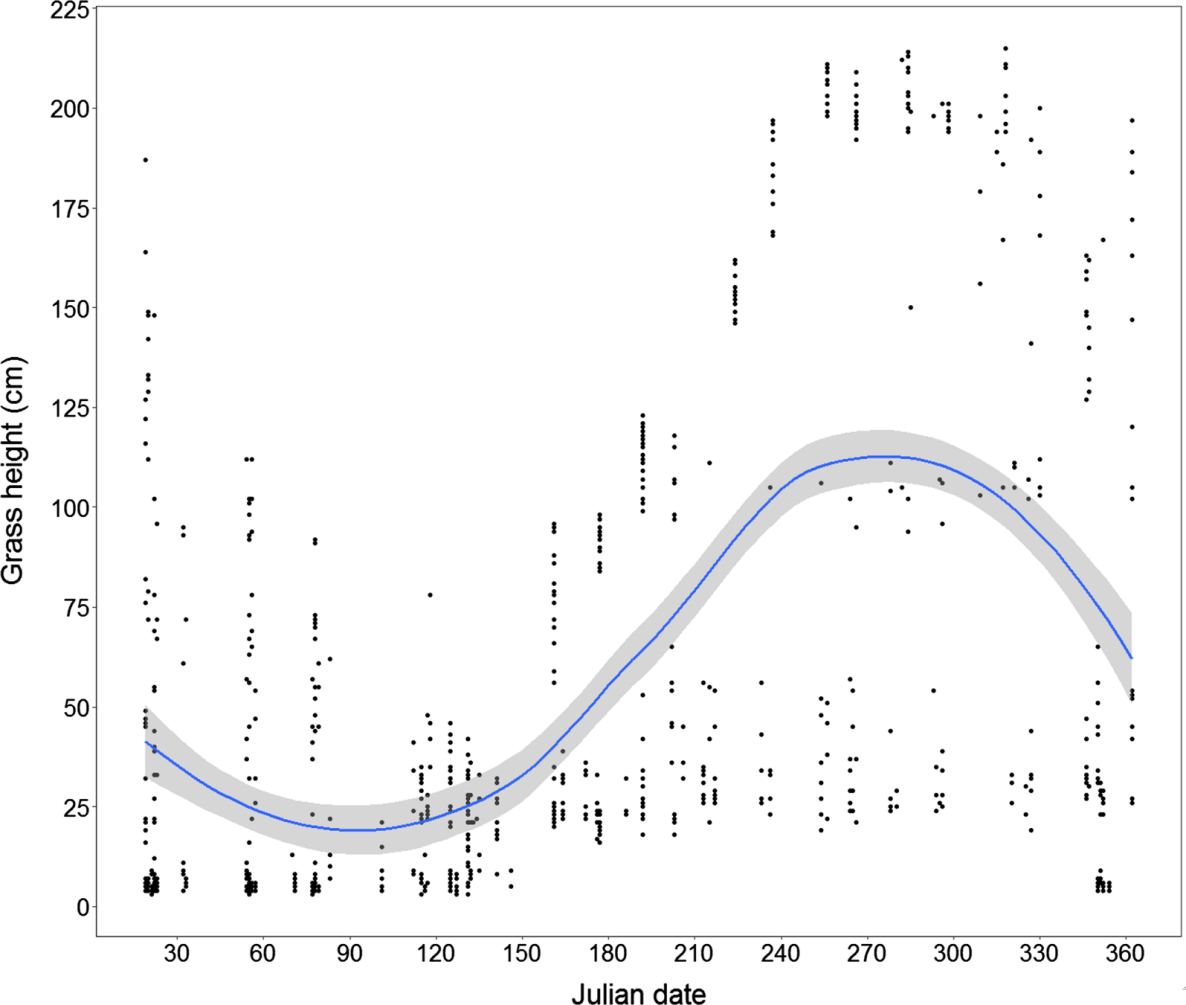
Grass height across Julian date in Maze National Park analyzed using a fixed effect model with 95% confidence interval in Maze National Park. The grass heights were randomly measured from random plots in each month for a year

The random grass height measurements in Maze National Park showed a significant increase of grass height with increasing time (Fig. 3).

**Fig. 4:**
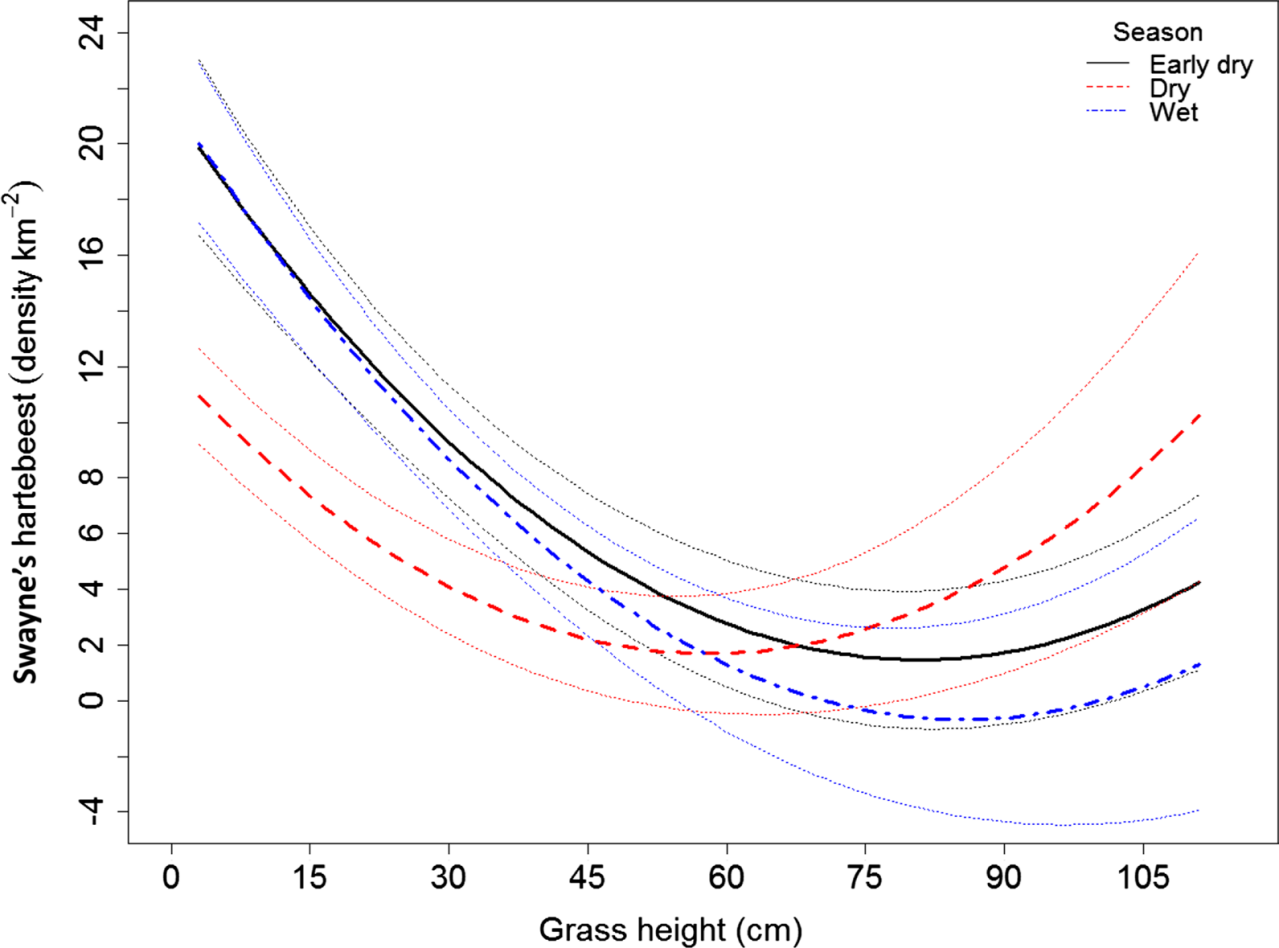
Predicting the density of Swayne’s hartebeest in relation to grass height preference in three seasons, namely Early dry (solid black line), Dry (dotted red line), and Wet (dotted blue line) in Maze National Park

During the dry season, we recorded 3225 grazing events while studying their grass height preference. Of this, 2288 (70.9%) individuals were recorded below 30 cm grass heights. The rest 540 (16.7%), 258 (8%) and 139 (4.3%) of grazing events were recorded between 31 and 50 cm, 51–100 cm and above 100 cm grass heights, respectively. During the wet season, we recorded 1266 grazing events. Of this, 1079 (85.2%) were recorded below 30 cm grass heights. The rest 156 (12.3%), 29 (2.3%) and 2 (0.2%) grazing events were recorded between 31 and 50 cm, 51–100 cm and above 100 cm grass heights, respectively. During the early-dry season, we recorded 695 grazing events. Of this, 593 (85.3%) were recorded below 30 cm grass height. The rest 78 (11.2%) and 24 (3.5%) grazing events were recorded between 31 and 50 cm and above 50 cm grass heights, respectively. Swayne’s hartebeests strongly preferred shortest available grass height in all seasons, with a decrease in the density of animals with increasing grass height (Table 3, Fig. 4). The decrease was stronger during wet and early-dry seasons compared to dry season. Areas with taller grasses are more used during the dry season than other seasons (Fig. 4).

**Table 3:** Estimates of Swayne’s hartebeest density in grassland habitat in relation to season and grass height in Maze National Park analyzed using general linear mixed-effects model

Effects	Estimmate	SE	t-value	p-value
Intercept	12.428	1.286	9.666	< 0.001
Dry season	− 2.174	1.107	− 1.963	0.050
Wet season	2.394	1.423	1.682	0.093
Grass height	− 0.102	0.018	− 5.556	< 0.001
Dry season × grass height	− 0.064	0.025	− 2.556	0.012
Wet season × grass height	− 0.100	0.034	− 2.906	0.004

Early-dry season was used a reference level for season categorical variable

### Impact of fire on Swayne’s hartebeest habitat use

**Fig. 5:**
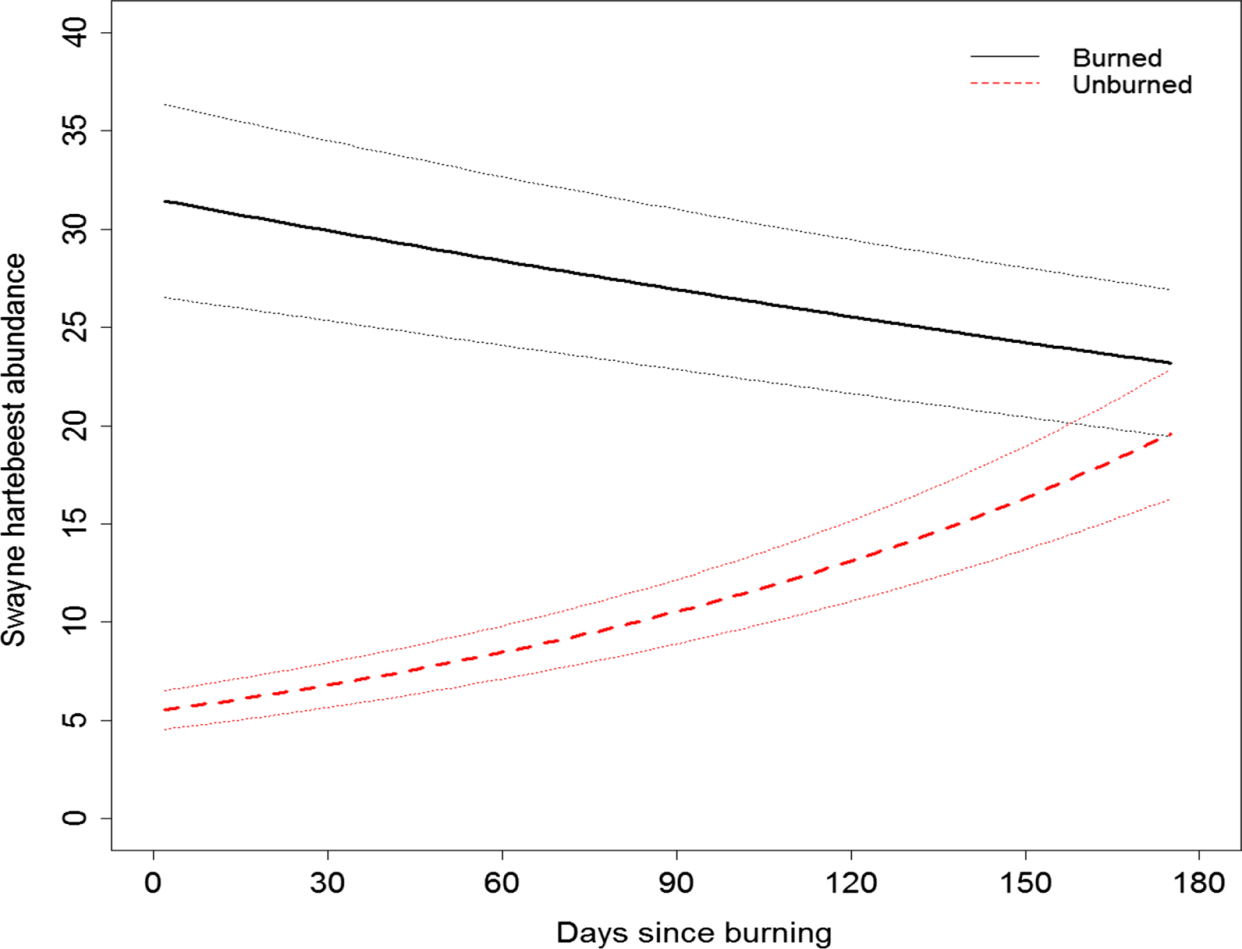
Predicted Swayne’s hartebeest abundance both burned (solid black line) and unburned (dotted red line) grassland areas in Maze National Park in relation to days since burning

Swayne’s hartebeest was attracted by burned grassland areas since the day of burning. In the first 30 days since burning, 54.5% of the observed Swayne’s hartebeests were found in burned grassland areas. From 31 to 60, 61–90, 91–120 and > 121 days since burning, we found 90.8%, 89.1%, 66% and 47.5% of individuals in burned grassland areas from the total observed Swayne’s hartebeests, respectively. The density of Swayne’s hartebeest in burned grassland area was significantly higher than unburned grassland areas up to 150 days after the initial burning (Table 4, Fig. 5).

**Table 4:** Swayne’s hartebeest abundance in grassland habitat in relation to fire disturbance (burned vs. unburned) in Maze National Park analyzed using generalized linear mixed effect model

Effects	Estimate	SE	z-value	p-value
Intercept	3.451	0.080	43.14	< 0.001
Un-burned vs. burned	− 1.754	0.058	− 30.41	< 0.001
Days vs. burned	− 0.002	0.000	− 6.07	< 0.001
Un-burned × days	0.009	0.001	16.97	< 0.001

## Discussion

Our study showed that Swayne’s hartebeests preferred open grassland habitat in Maze National Park throughout the year as observed with other wild herbivores, such as Coke’s hartebeest (*Alcelaphus buselaphus cokii*) in Athi-Kapiti Plains, Kenya [51]; hartebeests (*Alcelaphus buselaphus*) in southern border of Burkina Faso [52] and wildebeest (*Connochaetes taurinus*) in Serengeti National Park, Tanzania [9]. Although the Park has a wider coverage of other habitat types, such as bushland habitats and riverine forest, the Swayne’s hartebeest rarely used them. This reflects on the fact that Swayne’s hartebeest conservation is largely based on the management of the grassland habitat in Maze National Park. Our surveys detected few Swayne’s hartebeest pellets in bushland habitats and riverine forests during the dry season, which likely occurred when they were walking to a water source. Swayne’s hartebeests were not encountered in agricultural lands and rugged habitats except in a rare occurrence, which might have been a response to predators in the area.

In the grassland habitat, the grass grows fast and reaches above one m within a month after the wet season begins and becomes taller in the early-dry season, but decreases in height in the first few months of dry season (Fig. 3). However, Swayne’s hartebeests almost abandoned the taller grass height, and consistently preferred the shorter (below 30 cm) available grass height areas in the Park. Our findings are thus in support of the previous studies in other areas, for instance, hartebeests and roan antelope (*Hippotragus equinus*) in Nazinga Game Ranch, Burkina Faso [52], and wildebeest in Serengeti Park, Tanzania preferred short grass height [15]. There are two speculations about short grass preferences of herbivores: (1) due to the higher nutritional quality of short grasses and (2) to avoid predation risk. Even though the fear of predation may influence short grass habitat selection in some cases, in this study the predation risk is rather less due to low density of predators (mainly lions) in the Park (Misganaw et al. unpublished data), suggesting that the Swayne’s hartebeest preference of short grass habitats is more likely the result from nutritional gain. Shorter grasses have less lignin with lower carbon to nitrogen ratios which are more palatable and digestible for grazers [38]. Shorter grasses also have higher nutritional quality [31, 53] and percentage of green leaves that allow higher bite rates for herbivores foraging [28].

Grass height preferences of Swayne’s hartebeest influence their distribution in Maze National Park in different seasons. Previous studies [29, 54, 55] also revealed that forage influences the distribution of herbivores. The influence is demonstrated on the distribution of herbivore on its bite size [32]. During the wet season, Swayne’s hartebeest populations were concentrated in three small grassland patches over three months where the grass heights were shorter; this might be due to the soil type. Wildebeest herds in Kruger National Park, South Africa also concentrated in small grassland patches during the wet season where grasses were short and abundant [56]. Since the grass height in most parts of Maze National Park became above one m during the early-dry season, the Swayne’s hartebeests dispersed toward the periphery of the Park to find short grass sward that resulted in a significant difference of Swayne’s hartebeest density.

While a long-term study is needed to fully acknowledge on the use of fire as a management tool, our study suggests the annual fire is important for the conservation of the Swayne’s hartebeest in Maze National Park. This is evident following two facts: (1) Swayne’s hartebeests are highly attracted to burned grassland areas and (2) they avoid taller grasses. While fire destroyed much of the forage at the time of burning, field studies [42, 45] revealed that herbivores including hartebeest and wildebeest are attracted to burned grassland patches immediately after burning [42, 45]. There are four main speculations to explain the reason for herbivore attraction towards burned areas: avoiding parasites [10, 57], attraction by new flush grass shoots which are highly nutritious and easily digestible afterwards [15, 39], attraction by ash immediately after burning [58, 59] and detecting predators from far distances [15, 60].

The immediate use of burned areas by the Swayne’s hartebeest might be to get relief from parasites e.g. ticks and flies, which are commonly found in unburned grassland areas [57, 61]. For instance, burning grassland patches in Ngorongoro Crater, Tanzania during the dry season virtually eliminated tick populations which makes the area highly preferred by herbivores [57]. Another reason might be to acquire minerals from ash by licking the burned soil that are not obtained from available forage [58]. This is because ash is high in calcium (Ca), potassium carbonate (K2CO3), phosphate (PO4) and trace minerals content [58]. After few weeks of the burning time, however, the attraction of the Swayne’s hartebeest is not surprising due to the availability of fresh grass in burned grassland patches [14, 15, 39, 45]. The predation avoidance strategy in using open planes of burned area [15, 58] might not be a case for Swayne’s hartebeest in Maze National Park. From ad hoc observations we made during the study period, we encountered 13 carcasses of Swayne’s hartebeests; of these six were predated in the burned grassland habitats (Misganaw et al., unpublished data); suggesting that burned grassland area did not guarantee the Swayne’s hartebeest not being predated.

After 150 days of Swayne’s hartebeest attraction towards the burned area, the difference in use between the burned and unburned grassland areas diminishes possibly because both areas had similar grass height and nutrition content [14, 15]. Studies on Thomson’s gazelles (*Eudorcas thomsonii*) and impala (*Aepyceros melampus*) [15] reported similar trends that preferred fresh grasses in burned area over unburned green grass in the first months of post-fire in equatorial grassland ecosystem [14, 26, 27]. Further post-fire studies and vegetation monitoring is needed to understand the long-term effects of using fire as a management tool in Maze National Park.

## Conclusion

Swayne’s hartebeests in Maze National Park prefer grassland habitat and available short grass height throughout the year. This emphasises on the importance of the management of the limited grassland habitat available in the Park for the conservation of the Swayne’s hartebeest. The burned grassland patches in the Park strongly attract Swayne’s hartebeest starting from the next day of burning. They extensively use the burned grassland patches over the unburned areas up to when the two grassland areas have similar grass heights. This study suggests that controlled burning of the grassland areas in the Swayne’s hartebeest prime habitats may be an important habitat management practice. However, a long-term effect of burning and further details of the frequency and period of burning may help to substantiate our results.

## Supplementary information

**Additional file 1.** Map showing slope (degrees) gradient of Maze National Park.

**Additional file 2.** The map shows the Swayne’s hartebeests’ spatial patterns in different seasons in Maze National Park (MNP). **(A)** Depicts Swayne’s hartebeest presence points during dry season. **(B)** Depicts early-dry and wet seasons Swayne’s hartebeest presence points. The main road crosses the park and is leading from Wolaita Sodo to Gofa (Saula) towns. Roads (red lines) inside the park used for patrolling purpose. Scouts use Monoqo camp site to control the southern portion of the park.

## Data Availability

The datasets used and/or analyzed during the current study are available from the corresponding author on reasonable request.

## Abbreviations

CEES: Centre for Ecological and Evolutionary Synthesis
IUCN: International Union for Conservation of Nature
MNP: Maze National Park
EWCA: Ethiopian Wildlife Conservation Authority
GPS: global positioning system
