# Supplementary material for: The effect of season and post-fire on habitat preferences of the endangered Swayne’s hartebeest (Alcelaphus buselaphus swaynei) in Maze National Park, Ethiopia
Source: BMC Ecol. 2020 Jan 28;20:5. doi: 10.1186/s12898-020-0275-3 (PMC6986001; doi:10.1186/s12898-020-0275-3)
Supplement: Supplementary file 1 — Additional file 1. Map showing slope (degrees) gradient of Maze National Park. [file 12898_2020_275_MOESM1_ESM.pdf]

Additional file 1

Journal: BMC Ecology

Title: The effect of season and post-fire on habitat preferences of the endangered Swayne's hartebeest *(Alcelaphus buselaphus swaynei*) in Maze National Park, Ethiopia

Authors: Misganaw Tamrat, Anagaw Atickem, Diress Tsegaye, Paul Evangelista, Afework Bekele, Nils Chr Stenseth

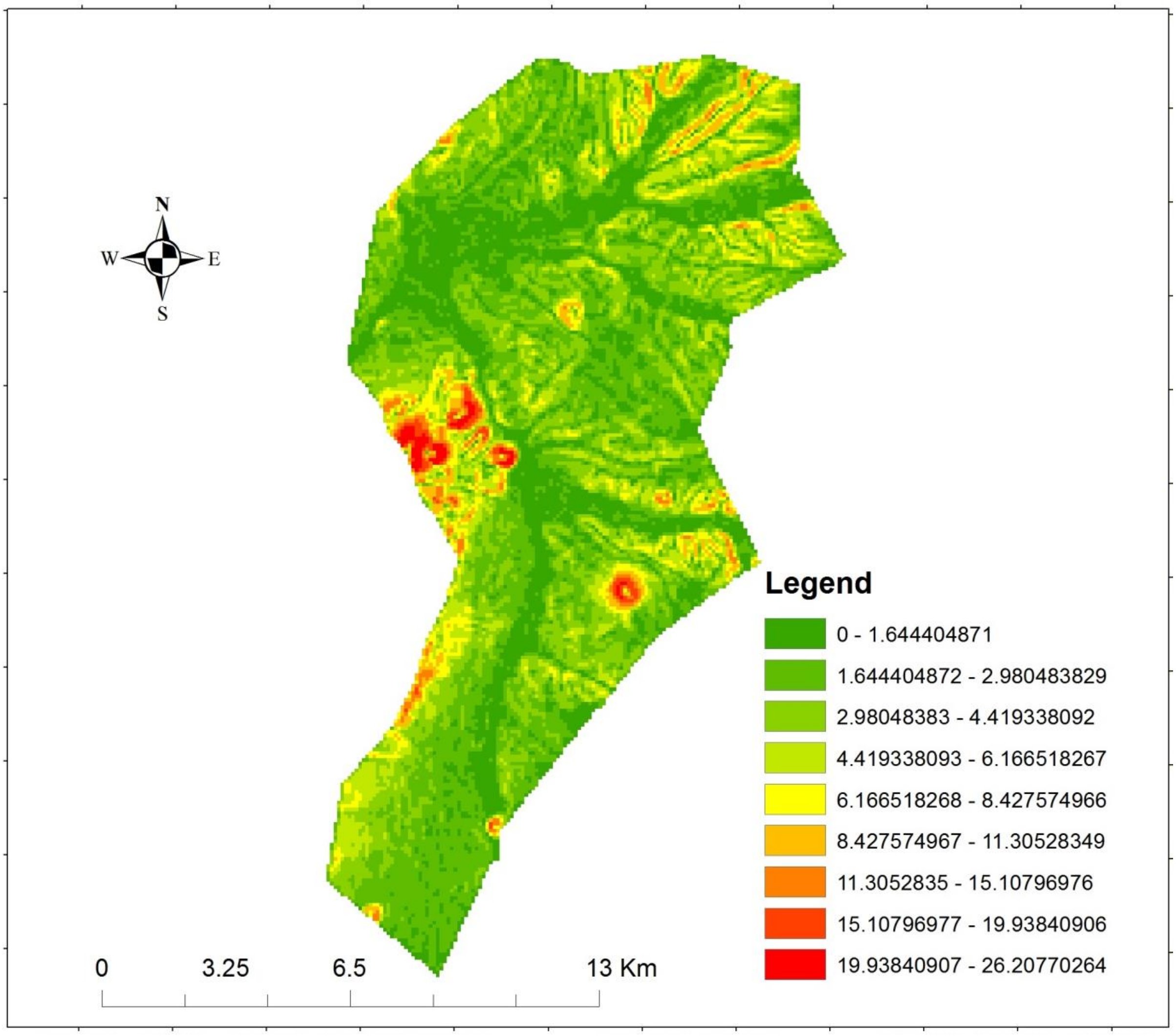

Additional file 1 Map showing slope (degrees) gradient of Maze National Park.
