# Supplementary material for: The effect of season and post-fire on habitat preferences of the endangered Swayne’s hartebeest (Alcelaphus buselaphus swaynei) in Maze National Park, Ethiopia
Source: BMC Ecol. 2020 Jan 28;20:5. doi: 10.1186/s12898-020-0275-3 (PMC6986001; doi:10.1186/s12898-020-0275-3)
Supplement: Supplementary file 2 — Additional file 2. The map shows the Swayne’s hartebeests’ spatial patterns in different seasons in Maze National Park (MNP). (A) Depicts Swayne’s hartebeest presence points during dry season. (B) Depicts early-dry and wet seasons Swayne’s hartebeest presence points. The main road crosses the park and is leading from Wolaita Sodo to Gofa (Saula) towns. Roads (red lines) inside the park used for patrolling purpose. Scouts use Monoqo camp site to control the southern portion of the park. [file 12898_2020_275_MOESM2_ESM.pdf]

Additional file 2

Journal: BMC Ecology

Title: The effect of season and post-fire on habitat preferences of the endangered Swayne's hartebeest (*Alcelaphus buselaphus swaynei*) in Maze National Park, Ethiopia

Authors: Misganaw Tamrat, Anagaw Atickem, Diress Tsegaye, Paul Evangelista, Afework Bekele, Nils Chr Stenseth

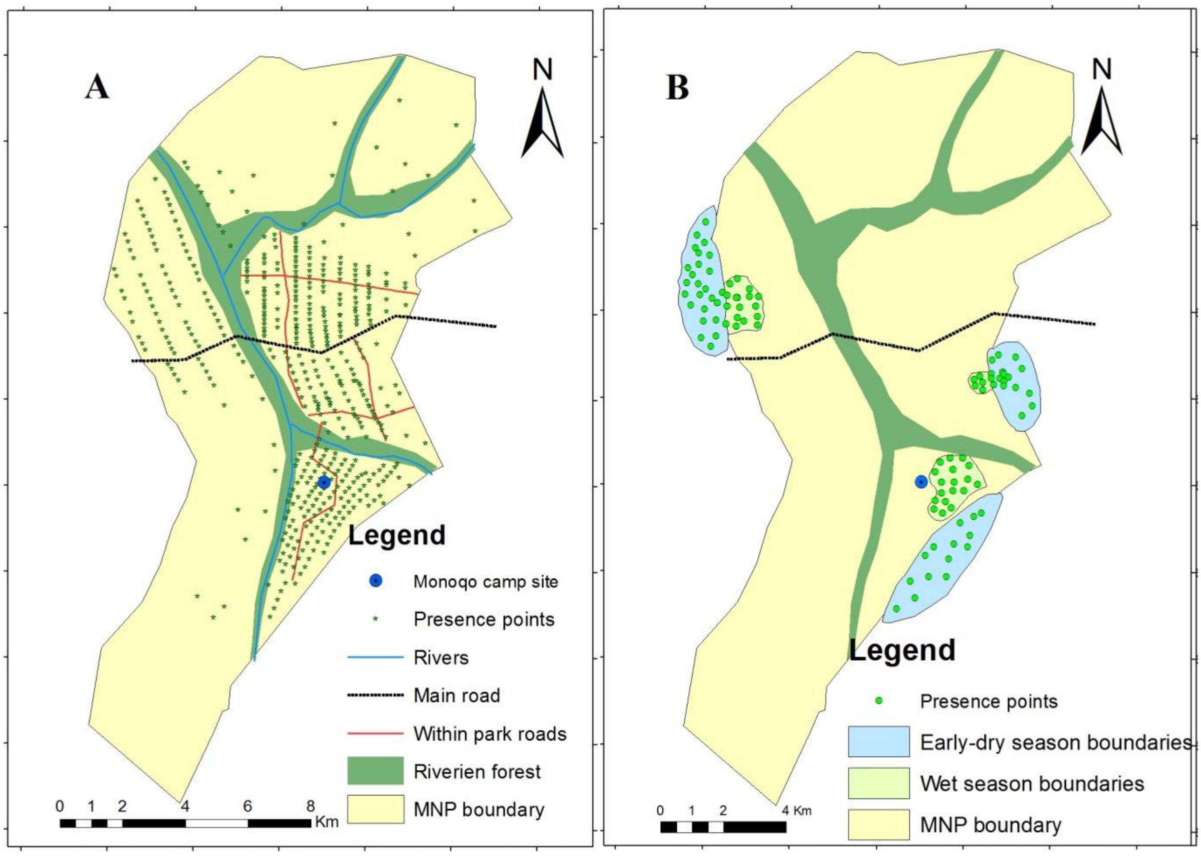

Additional file 2 The map shows the Swayne's hartebeests' spatial patterns in different seasons in Maze National Park (MNP). **(A)** Depicts Swayne's hartebeest presence points during dry season. **(B)** Depicts early dry and wet seasons Swayne's hartebeest presence points. The main road crosses the park and is leading from Wolaita Sodo to Gofa (Saula) towns. Roads (red lines) inside the park used for patrolling purpose. Scouts use Monoqo camp site to control the southern portion of the park.
